# The Platform Messaging Effect (PME): A quantification of how go-vote reminders on social media platforms can influence voting intentions

**DOI:** 10.1371/journal.pone.0343692

**Published:** 2026-03-02

**Authors:** Robert Epstein, Amanda Newland, Li Yu Tang, Alyssa Edmison

**Affiliations:** American Institute for Behavioral Research and Technology, Vista, United States of America; University of Amsterdam, NETHERLANDS, KINGDOM OF THE

## Abstract

Over the past decade, researchers have identified and quantified about a dozen new forms of influence made possible by the internet. Although experts have expressed concern about how users may be harmed by social media content, few studies have sought to quantify the extent to which go-vote reminders on such platforms can impact voting. In the present study, we sent go-vote reminders to US voters on a simulation of Facebook and found (a) that interspersing vote reminders in the message feed significantly increased the magnitude of voting intentions (by over 40% under certain conditions) and (b) that vote reminders also had a significant impact on how people might vote. Because reminders of this sort are ephemeral, social media platforms that send vote reminders to users in a partisan fashion might be able to shift the outcomes of close elections without people’s knowledge.

## 1. Introduction

Recent research has demonstrated the enormous power that online social media platforms, such as Facebook and X (f.k.a., Twitter), have to influence the emotions, opinions, and behavior of users on- and off-line [1–3], either because posted content is biased or “contagious” [1,2,4–6] or because of social pressure [7–11]. Social media platforms can have especially widespread and rapid effects on political opinions and behavior [7–22]. In the present paper, we will not be examining the power that social influence has on social media platforms; rather, we will be addressing a narrow issue, namely: to what extent might messaging from the platform itself – in other words, from the company that controls the platform – impact the outcome of elections?

About a dozen new methods of influence have been discovered, studied, and quantified over the past decade which give large online platforms unprecedented power to alter people’s thinking and behavior – especially people who are undecided on some issue – without their knowledge and without leaving paper trails for authorities to trace. Among these techniques are the “search engine manipulation effect” (SEME) [23; cf. 24–32], the “search suggestion effect” (SSE) [33], the “answer bot effect” (ABE) [34], the “video manipulation effect” (VME) [35], and what might be called the “predicted text effect” (PTE) [36]. Related research on the “digital personalization effect” (DPE) has shown that when content is personalized, it can substantially increase the impact of such techniques [37]. Other research on the “multiple exposure effect” (MEE) has also shown that the impact of repeating the same technique on one platform can amplify the impact of a single exposure [38], and a recent study on the “multiple platforms effect” (MPE) has shown that the impact of similarly biased content presented to users on different platforms can also amplify the impact of a single exposure [39].

In the present paper, we are investigating an effect we call the “platform messaging effect” (PME), which we define as the potential impact that platform content has on the thinking and behavior of users when that content has been sent directly from the company that owns and controls the platform. As we noted above, we believe that content of this sort needs to be studied in its own right because (a) it cannot be counteracted by third parties, (b) unlike organic content posted by users, it is ephemeral by nature and therefore leaves no paper trail for authorities to trace, (c) it can, based on the large profiles of information the company has collected about its users, be personalized to maximize its impact (for example, it can be directed at users who are undecided on an issue and who are therefore especially vulnerable to influence) [cf. 39], and (d) it can be presented in ways that minimize users’ awareness that they are being manipulated.

To be clear, we will not be examining how political campaigns, political action committees, or foreign actors might use messaging on social media platforms to influence the outcomes of elections. This can be done through targeted advertising [13–17], the unleashing of thousands of bots [40–42], announcements coming from fake organizations [43,44], and fake content (such as “deepfakes”) made with artificial intelligence software [45,46]. We are deliberately setting aside forms of influence of this type because they are inherently competitive and therefore pose no inherent threat to the integrity of the free-and-fair election [cf. 40–42]. Influence from competitive sources such as political campaigns is comparable to legacy forms of influence: billboards, television and radio advertisements, and so on [47–51]. To this day, if one party uses a billboard to support its candidate, the other party can buy a billboard (or two, or even 10 billboards) to support the opposing candidate. This competitive process applies to every new form of messaging a campaign might employ on a social media platform. It even applies to techniques used by bad actors; if Russian hackers use bots to post thousands of messages on Facebook to support one candidate in a US election, their impact could be nullified in a number of ways. For example, Facebook might be able to identify and remove the suspect content; the campaign organization supporting an opposing candidate might use bots to post millions of their own messages; that campaign organization might also choose to expose the Russian plot, thus shaming the opposing candidate.

But influence that is coming directly from the company-owned platform (say, from Meta, the company that owns Facebook) is inherently non-competitive. The platform itself can not only post vast numbers of messages, it can also target those messages to maximize their impact [13,14,42,52]. And whereas Facebook’s customers have to pay considerable sums to post their ads [53], the platform itself can continue to post consequential, targeted messages for long periods of time at no additional cost to the company. What’s more, whereas messages on billboards or posted by bots usually have an obvious political bias, the platform itself can post messages that are structured and targeted in ways that obscure their manipulative intent – in other words, in ways hide that intent from users [23,33,34,37–39,52,54,55]. We will return to this issue in our Discussion.

In addition to sending out influential messaging, platforms such as Facebook, Google, Instagram, TikTok, and Amazon can also shift people’s thinking and behavior by strategically filtering the content they display – showing content that favors one brand or one political candidate, for example – and by strategically ordering such content so that one brand or one candidate appears higher in lists. One such study, published by Facebook employees and academic collaborators in 2014, showed that deleting organic content that contained heightened emotional language (emotionally positive language for one group, emotionally negative language for a second group) produced a corresponding change in the emotional language of users who viewed that content [2]. Perhaps because of strong criticisms expressed about this study – especially about the fact that the manipulation was exercised without the consent or knowledge of users [56–59] – since the Kramer et al. (2014) study was published, neither Facebook nor any other major tech platform has, to our knowledge, admitted to using similar manipulations.

The dramatic impact that filtering and ordering can have on thinking and behavior has been studied most extensively with search engine results [23; cf. 24–32], but it has also been documented with product displays [60–62] and search suggestions [33]. The flip side of filtering and order to favor one perspective is what many people would call “censorship”: demoting or deleting content that favors one perspective [63,64]. Social media platforms sometimes justify the deletion of content by labeling it “hate speech” or “misinformation,” but people tend to differ in how they use such labels [65]; the deletion of what some people consider “hate speech” might be viewed as a violation of free speech rights by others [65,66]. In the US, these practices – the strategic filtering, order, demoting, and deleting of content – are generally allowed by courts, which have compared such practices to the editorial practices of newspaper editors [67,68, cf. 66,69]. US Courts have also ruled that online platforms can show any content they like under the protection of the First Amendment to the Constitution [67,70].

Whistleblowers and leaked documents from Google, Facebook, and X (formerly Twitter) have confirmed that these companies often manipulate the thinking, emotions, and behavior of users deliberately – sometimes, as we have seen above, simply to experiment, but also to impact values and political thinking [71], increase watch time [72,73], or increase revenues [73]. It is beyond the scope of this paper to review the growing and extensive literature on how tech companies appear to be using their new powers of influence. Instead, as noted above, we will focus on just one issue that we believe deserves close study: how these companies might be able to determine the outcomes of elections using direct messaging they insert into their content feeds.

Although most studies of influence on social media platforms have focused on the influence of organic content posted by users or bad actors, one study, along with a replication study published a few years later, looked at the impact of content posted by the platform itself. That study, published in *Nature* by Robert M. Bond, a political scientist at the University of California San Diego (UCSD), along with four other UCSD faculty members and two data scientists from Facebook, described an experiment in which Facebook sent go-vote reminders to 61 million Facebook users on Election Day in the 2010 midterm elections in the US [7]. The authors concluded that their go-vote messages likely caused 340,000 people to vote that day who otherwise would have stayed home. They also concluded that their messages “not only influenced the users who received them but also the users’ friends, and friends of friends” [7, p. 295]. The published study showed the go-vote image Facebook had posted (S1 Fig). It concluded, “The results show that the messages directly influenced political self-expression, information seeking and real-world voting behavior of millions of people” [7, p. 295]. In 2017, Bond, along with two other academic researchers and two Facebook employees, published a replication of the 2012 study. In the replication, go-vote reminders posted on the home pages of 15,060,897 Facebook users on Election Day in the Presidential election of 2012 appeared to cause a total of 270,000 more people to vote that day [9]. As far as we are aware, these are the only two published studies that look directly at what we’re calling the “platform messaging effect” (PME), and the second is a replication of the first. In our view, these studies are limited in two important ways.

First, one might ask, why aren’t there more studies that look directly at what happens when a platform sends its own messages – as opposed to organic content or advertisements – directly to its users? The simple answer to this question is that both of these studies were collaborations between university researchers and Facebook personnel, two of whom were listed as co-authors on the original publication and two others on the replication [7,9]. As far as we are aware, no independent researchers have replicated their findings. Generally, it appears that the leaders of tech companies are not anxious to expose the power they have to influence their users. What’s more, how might researchers test this effect in a laboratory setting without direct access to the platform or its users? We believe the best way for independent researchers to do so is by creating a simulation of Facebook, the method we used in our experiment.

Second, because the Bond studies were conducted as field experiments, the researchers were limited in the level of control they could exert over their variables. Can employing a pre- and post-test experimental design provide a clearer picture of the specific mechanisms that make vote-reminders effective at increasing voting intentions? Moreover, are voting intentions the only factor that vote reminders can influence? The present study addresses these questions.

### 1.1 Social media simulators

Unless independent researchers have direct access to company platforms, they have no way to determine the full extent to which platform content can influence users. In the absence of such access, researchers have relied on simulations of company platforms to study influence. Using close simulations of the software and representative samples of users, researchers can at least approximate the power tech companies have to influence thinking, emotions, and behavior. Note that simulations only measure possibilities; without confirmation from company officials, whistleblowers, or leaked or subpoenaed content, one cannot know whether and how new techniques of influence are being employed. In our Discussion section below, we will also show how “passive monitoring systems” can be used to track content being sent to users; such systems can determine whether certain methods of influence – even methods that are invisible to users – are being used.

Several recent studies show how different types of simulations are currently being employed to study online influence. For example Liu et al. (2021) created a micro-blogging website that featured elements of both Twitter and Facebook to compare the effectiveness of “expressions” (“active engagement of users with content, such as likes and comments”) versus “impressions” (“exposure to content, such as views”) on the views of 287 people recruited from the Amazon Mechanical Turk (MTurk) subject pool [74]. 200 of those people were instructed to post content intended to influence people’s views on eight current and controversial topics (e.g., “Is the US doing enough to combat COVID-19?”) – these were the “expressions” – and the remaining 87 participants just viewed content being posted – thus receiving “impressions.” The researchers found that impressions had a significantly greater impact on people’s view than expressions did.

In a more recent study, researchers posted an open-source social-media-like website called “The Misinformation Game” to help researchers study how misinformation affects people’s opinions [75, cf. 76, 77]. Simulations are helpful in research because they allow researchers to control a wide variety of platform features, in this case “posts (e.g., headlines, images), source information (e.g., handles, avatars, credibility), and engagement information (e.g., a post’s number of likes and dislikes).”

Since 2013, Robert Epstein and his associates have been discovering, studying, and quantifying new means of influence made possible by the internet using simulations of the Google search engine [e.g., 23, 37, 38], Google search suggestions [e.g., 33], YouTube [e.g., 35], the Alexa personal assistant [e.g., 34], Twitter [e.g., 52], and other online platforms [e.g., 54].

In the present study, we aim to answer the following question: To what extent might go-vote reminders inserted into a social media feed by the company that owns the platform impact the voting intentions of its users? To answer this question we designed a randomized, controlled, counterbalanced, double-blind experiment that utilized a close simulation we built of the Facebook platform.

In the Facebook simulation, we exposed some participants to vote reminders announcing a special election; the reminders appeared to come from the company itself. Before and after exposure to the simulation, we measured three important factors: (a) the participants’ awareness of the special election, (b) the participants’ intent to vote in the special election, and (c) the participants’ attitude regarding that issue raised in the special election. We then analyzed the data we collected for pre-to-post shifts across all three measures to determine the extent of the impact of the go-vote reminders. We predicted that as a result of our manipulation, we would find an increase in both awareness and intent to vote in the special election for the participants who saw the vote reminders. We also predicted that our reminders would likely *not* affect people’s attitudes regarding issues.

Please note that our experimental design only allowed us to measure our participants’ intent to cast a vote. Past research has shown how voting intentions do not always align with other voting behaviors, including actually casting a vote or reporting having done so [78–80]. We will talk more about this issue in our Discussion section.

## 2. Methods

### 2.1 Ethics statement

The federally registered Institutional Review Board (IRB) of the sponsoring institution (American Institute for Behavioral Research and Technology) approved this study with exempt status under HHS rules because (a) the anonymity of participants was preserved and (b) the risk to participants was minimal. The IRB is registered with OHRP under number IRB00009303, and the Federalwide Assurance number for the IRB is FWA00021545. Informed written consent was obtained for the experiment as specified in the Procedure section below.

### 2.2 Participants

Participants were recruited online from the Amazon Mechanical Turk (MTurk) subject pool between February 10th and February 27th, 2025. Participants were screened by CloudResearch to prevent bots and suspect users from entering our subject pool. Participants were allowed to participate only if they answered Yes to the question, “Are you eligible to vote in the United States?”

Before cleaning we had data from 594 participants. We removed participants who reported an age lower than 18 or who reported residing outside of the US. We also asked participants to report their English fluency on a scale from 1 to 10, where 1 was labeled “Not fluent” and 10 was labeled “Highly fluent.” We removed participants who reported a fluency level below 6. After cleaning we had data from 592 participants. To equalize the size of the two groups we analyzed, we used SPSS to draw at random (and without replacement) the largest possible sample we could obtain from each of the two groups, giving us 267 people in each. In total, therefore, we analyzed data from 534 people in this experiment.

The participants ranged in age from 19 to 77 (*M* = 45.3, Median = 43, *SD* = 12.5). 48.5% (*n* = 259) of the participants identified themselves as female, 51.1% (*n* = 273) as male, and 0.4% (*n* = 2) did not identify their gender. 81.1% (*n* = 433) of the participants identified themselves as White, 7.9% (*n* = 42) as Black, 4.1% (*n* = 22) as mixed, 4.7% (*n* = 25) as Asian, and 2.2% (*n* = 12) as other. Regarding the level of education people reported having completed, 0% (*n* = 0) said none, 6.4% (*n* = 34) said primary, 31.8% (*n* = 170) said secondary, 42.9% (*n* = 229) said bachelor’s degree, 14.8% (*n* = 79) said master’s degree, and 4.1% (*n* = 22) said doctorate. 46.1% (*n* = 246) of the participants identified themselves as liberal, 24.2% (*n* = 129) as moderate, 26% (*n* = 139) as conservative, 2.6% (*n* = 14) as none, and 1.1% (*n* = 6) as other. 78.7% (*n* = 420) of participants stated that they had used social media like Facebook before, and 21.3% (*n* = 114) of participants stated that they had not.

### 2.3 Procedure

Participants were given brief instructions and were asked for their consent to participate in the study (see S2 Fig). They were then asked a series of basic demographic questions, after which they were given basic information about special elections and bond referenda (S3 Fig). Then, without their knowledge, participants were assigned at random to either the experimental group or the control group. In both groups, they were all asked the same three questions both before and after the manipulation, as follows:

1. “Have you heard about the upcoming special election in your state scheduled for later this year?” Participants clicked “Yes,” “No,” or “Not sure.”
2. “Do you think you will vote in the upcoming special election in your state scheduled for later this year?” Participants clicked “Yes,” “No,” or “Not sure.”
3. “In the upcoming special election, if you had to vote on a bond referendum, how would you vote?” Participants clicked “For,” “Against,” or “Not sure.”

In both groups, after these three pre-manipulation questions were asked, we gave participants brief instructions about how they should use “Doodlebook,” our social media platform, which is a close simulation of Facebook. The instructions were in part: “On the next page, you have the opportunity to scroll through posts on our simulated version of the Facebook social media platform.... You will see posts people have made about recent special elections held in many different states across the US. Please scroll through the entire... feed to gather information about what special elections are for” (for complete instructions, see S4 Fig). These instructions served as a distractor task. In reality, our participants would not be asked to explain why special elections are held.

Now, in the Control group, participants could scroll through 30 typical Facebook-type posts, all of which were composed by the researchers and all of which were about various special elections. Note that the posts were presented in a random order for each participant, and that none of these posts asked or encouraged people to vote in an upcoming special election. Adding political content to the typical Facebook posts added credibility to our distractor task and avoided causing confusion among the participants in the Control group.

**Fig 1:**
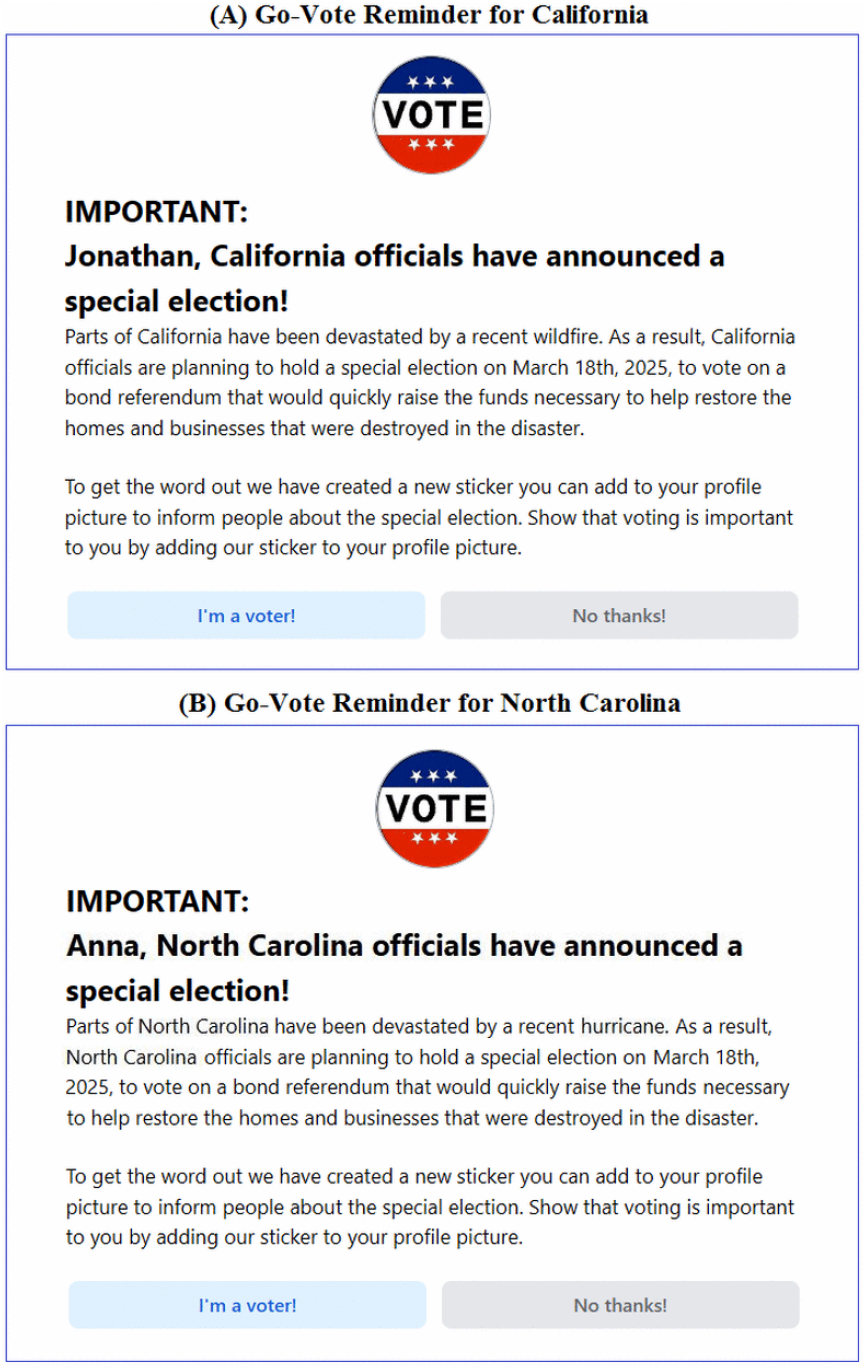
Pop-up Go-Vote reminder. **(A)** Example of the pop-up go-vote reminder seen by participants who reported living in California. **(B)** Example of the pop-up go-vote reminder seen by participants who reported living in North Carolina. The pop-up go-vote reminders were personalized to the participants using their name and the state that they live in. The natural disaster mentioned in the pop-up was the most recent natural disaster to happen in each state according to FEMA.gov.

**Fig 2:**
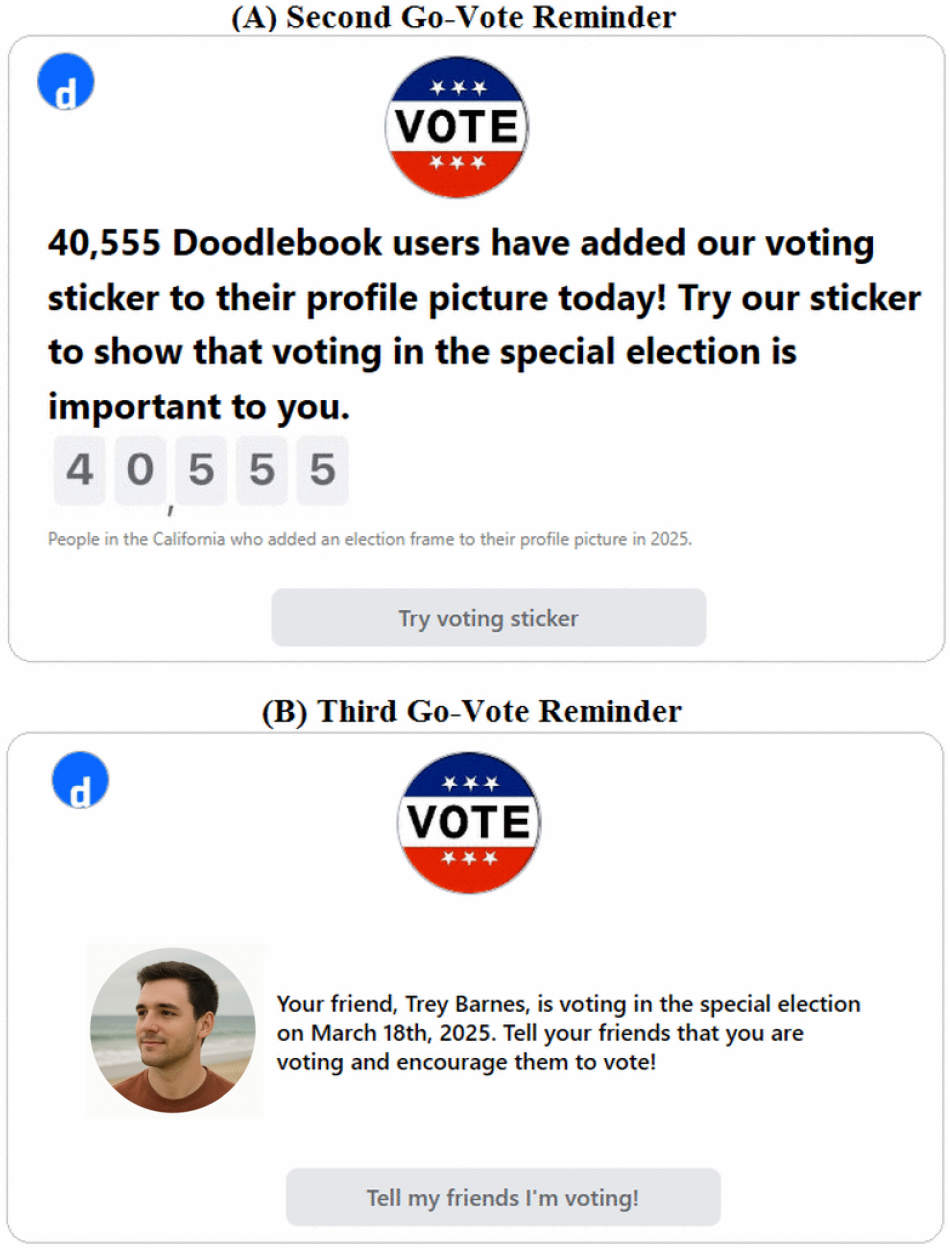
Second and third Go-Vote reminders. **(A)** Screenshot of the second go-vote reminder placed in the 11th position of the Doodlebook feed. **(B)** Screenshot of the third go-vote reminder placed in the 22nd position of the Doodlebook feed.

Participants in the Experimental group saw the same 30 posts as seen in the Control group, again in a random order for each participant. However, they also saw three additional posts, each one a go-vote reminder. The first was a pop-up that appeared just as the Doodlebook list appeared; the pop-up showed content that was specific to the user’s state (see Fig 1A and 1B for examples). The second and third posts were inserted into positions 11 and 22 and were the same for all users (see the two go-vote reminders in Fig 2).

The reminders shown to participants in the Experimental group were designed to mimic reminders shown to effectively impact voting behavior in past research [7,9], as well as reminders that were sent to real voters during the 2024 US Presidential Election (S5 and S6 Fig). Our reminders prompted participants to show that they were voting in the upcoming special election by adding a VOTE sticker to their Doodlebook profile picture. The reminders also mentioned how many other people in their state and which of their friends on Doodlebook had added the sticker. These features of the reminders attempted to appeal to our participants’ sense of civic duty and paint voting as a social norm. Past research has show that these tactics are effective in influencing voting behavior and these tactics are commonly used by social media companies in real go-vote reminders [81].

Participants were required to scroll through the feed for at least one minute before a grey “Submit” button appeared in the top right corner of the Doodlebook page. When participants clicked that button, they were taken to the next page, where they were asked those three voting-related questions again (shown above). Finally, participants were asked, “While you were conducting your research, did you notice anything that bothered you in any way?” They could respond Yes or No. If they responded Yes, they could give us further details in a text box. We asked this question to see if participants in either group felt manipulated in some way. We could not ask about manipulation directly because leading questions of this sort have long been known to inflate answers artificially [82].

Participants were debriefed on the last page of the session. They were thanked for their participation, given an MTurk code they could use to collect their payment, and given an email address where they could submit questions about the study or request that their data be removed from the study. Participants were also informed that all of the content they viewed in the study was created by the researchers and that the special election and natural disaster mentioned in the posts were not based on real events (see S7 Fig for the statement used to debrief the participants after the experiment).

### 2.4 Statistical analysis plan

To evaluate the effectiveness of the go-vote reminders in our simulation of Facebook, we calculated the pre- to post-manipulation percent increase (*P*) in the number of people answering our three multiple-choice voting questions in the affirmative. For example, if before scrolling through the Doodlebook feed, a total number of x participants said that they were planning to vote in the upcoming election, and if, after scrolling through the Doodlebook feed, a total of $x^{\prime }$ participants said that they were planning to vote in the upcoming election, $P=\text{1}\text{0}\text{0}*(x^{\prime }-x)/x$.

We used a McNemar’s chi-square test to evaluate the statistical significance of *P* for the experimental group and the control group. To compare *P* across the two groups, we employed a z-test. For all the statistics reported, we followed the standards of the current *Publication Manual of the American Psychological Association* [83] and reported the actual *p* value when that value was equal to or larger than .001; when the value was below .001, we reported *p* < .001. We used two-tail tests throughout.

## 3. Results

For the first voting question (“Have you heard about the upcoming special election in your state scheduled for later this year?”), in the Control group, the number of people responding “Yes” before entering the Facebook simulator was 44. After exposure to the simulator, that number increased by 13.6% to 50. That increase was not statistically significant (Table 1). In the Experimental group, the number of people who said that they had heard of the upcoming special election before entering the Facebook simulator was 55. After exposure to the simulator, that number increased by 138.2% to 131, a statistically significant increase (Table 1). The percent increase in the Control group and the percent increase in Experimental group for this measure were also significantly different from each other (Table 1).

**Table 1:** Pre-to-post-manipulation percent increase in number of people who had heard of the special election by group.

Group	*n*	Percent Increase	McNemar χ^2^	*p*
**Control**	267	13.6%	1.79	.18 NS
**Experimental**	267	138.2%	68.60	<.001
***z***		47.60		
***p***		<.001		

For the second voting question (“Do you think you will vote in the upcoming special election in your state scheduled for later this year?”), in the Control group, the number of people responding “Yes” before entering the Facebook simulator was 146. After exposure to the simulator, that number increased by 6.8% to 156. That increase was marginally significant (Table 2). In the Experimental group, the number of people who said that they were planning to vote in the upcoming special election before entering the simulator was 141. After exposure to the simulator, that number increased by 14.9% to 162, a statistically significant increase (Table 2). The percent increase in the Control group and the percent increase in Experimental group for this measure were also significantly different from each other (Table 2).

**Table 2:** Pre-to-post-manipulation percent increase in number of people who were planning to vote in the special election by group.

Group	*n*	Percent Increase	McNemar χ^2^	*p*
**Control**	267	6.8%	4.05	.04
**Experimental**	267	14.9%	12.90	<.001
***z***		4.26		
***p***		<.001		

For the third voting question (“In the upcoming special election, if you had to vote on a bond referendum, how would you vote?”), in the Control group, the number of people responding “For” before entering the Facebook simulator was 64. After exposure to the simulator that number increased by 1.6% to 65. That increase was not statistically significant (Table 3). In the Experimental group, the number of people who said that they would vote in favor of a bond referendum before entering the simulator was 68. After the simulator, that number increased by 51.5% to 103, a statistically significant increase (Table 3). The percent increase in the Control group and the percent increase in Experimental group for this measure were also significantly different from each other (Table 3).

**Table 3:** Pre-to-post-manipulation percent increase in number of people who would vote in favor of a bond referendum by group.

Group	*n*	Percent Increase	McNemar χ^2^	*p*
**Control**	267	1.6%	0.00	1.00 NS
**Experimental**	267	51.5%	21.81	<.001
***z***		18.46		
***p***		<.001		

Regarding the perception of our manipulation, only 2.6% (*n* = 7) of participants in the Experimental group said that the go-vote reminders bothered them. These participants described the reminders as “annoying” and “intrusive,” and said they felt “pressured” to add the vote sticker to their profile.

## 4. Discussion

### 4.1 Major findings

Our findings suggest a clear answer to the question we posed earlier: To what extent might messaging from a social media platform itself – in other words, from the company that controls the platform – impact the outcome of elections?

Our results suggest that repeated go-vote reminders sent to users on a popular social media platform in the days leading up to an election could increase voting intentions by 14.9%. The effect we found was larger than the effects found in field studies on the impact of go-vote reminders on voting behavior [7,9], perhaps because in a lab study we could better isolate and control our variables [84,85]. It could also be because our reminders used social pressure to persuade the participants, which, as we discussed in our Introduction, has been shown to be effective in altering voting behavior [7–11].

By making some reasonable assumptions, we estimate that in a national election in the US in which 154 million people were expected to vote, between 2 and 3 million additional people might vote if a widely-used social media platform repeatedly sent out go-vote reminders (See S1 Text and S2 Text for how we arrived at this estimate). This assumes that the platform in question is being used by between 60% and 70% of the voting population. In fact, Facebook is used by approximately 68% of the voting population of the United States [86]. Other popular platforms, such as Instagram and TikTok, are used by approximately 47% and 33% of eligible US voters, respectively [86].

Our results also showed that go-vote reminders have the power to influence how people will vote. We found a 51.5% increase in the number of people voting in favor of a bond referendum after viewing our go-vote reminders. This finding suggests that if those reminders were biased in a different way – say they were biased to favor one particular political candidate – that candidate might conceivably receive millions of additional votes in a national election – enough, perhaps, to change the outcome of the election.

Our speculations might be inaccurate – far too high, perhaps – but we ask the reader to bear in mind three factors that might greatly increase the impact of vote manipulations of the sort we have examined in the present study: First, we note that, unlike billboards and television commercials, platform manipulations (such as go-vote reminders inserted into organic feeds) cannot be counteracted by opposing parties; they are controlled completely by the platform executives or owners. Second, platforms can repeat these manipulations dozens or hundreds of times in the weeks leading up to an election, and it has been shown that the impact of repeating similarly biased content on a single platform tends to be additive [38]. And third, most social media platforms in the US tend to lean in the same direction politically [87]; if most or all of these platforms send similarly biased go-vote reminders to users, it is likely that the impact of these prompts will be additive [39]. Bear in mind that two of the largest social media platforms in the world – Facebook and Instagram – are owned by the same company (Meta).

Our study raises an interesting question. Would an online platform be so bold as to send out targeted, partisan go-vote reminders to its users in the days leading up to an election? Wouldn’t they risk exposure in the media? Unfortunately, platforms can send people partisan, targeted vote reminders in ways that are unlikely to attract the attention of voters, journalists, or authorities. One way is to send everyone vote reminders but to send them at different rates based on people’s political leaning. Even when members of only one party receive vote reminders, people are unlikely to suspect they are being manipulated; the voters who receive no vote reminders have no reason to suspect foul play. And if vote reminders are being sent *mainly* to members of one party, people are even less likely to spot the manipulation.

Moreover, to take action on any partisan manipulations of this sort, one would need to provide physical evidence that proves a social media platform sent go-vote reminders in a partisan fashion. Since the vote reminders are ephemeral, in order to prove to a court that a wrong has been committed, one would have to record the appearance and non-appearance of the reminders on a large number of computers before the relevant screen images disappeared from people’s screens. Word of mouth would not be enough to stop the manipulation. In fact, every type of go-vote manipulation we mentioned above has been documented in recent elections in the US using a growing nationwide monitoring system, which currently preserves and analyzes personalized ephemeral content being sent to the computers of a politically-balanced group of more than 17,000 registered voters in all 50 states (with voters’ permission). For details on monitoring systems, see [81].

### 4.2 Limitations and future research

Our study suffers from a number of limitations, perhaps the most obvious being that we tested go-vote reminders on a simulation of only one social media platform, Facebook. Additional research is necessary to determine the extent to which our findings are applicable to other popular platforms, such as Instagram, TikTok, X (f.k.a Twitter), and so on. Testing the effects of go-vote reminders on different platforms might be especially important for revealing how different platforms might influence different demographic groups differently. Younger voters are more likely to use Instagram or TikTok than Facebook to get political news [86,88]; is platform-driven messaging on Instagram or TikTok especially powerful in impacting the views of young people? In addition to testing go-vote reminders on different platforms, future research should also investigate the effectiveness of other kinds of political messages sent by social media companies, such as, register-to-vote reminders and mail-in-your-ballot reminders. A sequence of messages sent in a partisan fashion in the months leading up to an election – register-to-vote prompts, followed by mail-in-your-ballot prompts, followed by go-vote prompts – could presumably have a substantial effect on the outcome of the election.

Future research should also investigate how the number of go-vote reminders in a feed affects the size of PME. In the present study, we have three go-vote reminders in our feed. Could an effect still be found with a single message, as past research suggests [52]? Is there a maximum number of vote-reminders that can be sent before the majority of people get annoyed or the manipulation is no longer invisible?

We also worry about the ways the participants we obtained from MTurk might differ from the population of eligible voters in the US. On this issue, we can only note that we took the precaution of using a third-party company to screen out bots and suspicious users.

Lastly, we note that we did not measure actual voting behavior; we only measured three ways in which people’s *intentions* to vote had changed. Future research should explore how go-vote reminders on social media platforms might influence other kinds of political behavior. In particular, to what extent might the shifts we found in voting intentions be predictive of shifts in actual votes? Polls are often (but not always) predictive of actual voting patterns [89,90], but it is not clear to what extent the kinds of opinions we measured might be predictive.

The findings of the present study should not be overinterpreted. With a sample of 534 eligible US voters, we have shown that go-vote reminders can significantly shift voting intentions in predictable ways. But our participants were not real voters receiving information about a real election. As we mentioned at the beginning of this article, the internet has made possible dozens of new forms of influence, and in a real election online users might be subjected to multiple, if not all, of the new forms of influence that have the potential to impact their voting decisions. That said, we note three reasons why we believe PME should be a cause of concern for those worried about the integrity of our elections.

First, PME is a largely invisible manipulation. Only 2.6% of our participants mentioned concerns related to the go-vote reminders on our simulated Facebook feed. As we mentioned in our Introduction, messages generated by social media platforms themselves are often structured and targeted in ways that obscure their manipulative intent. The subtle nature of these manipulations allow people to be influenced without their awareness and lead people to mistakenly believe that they have made up their own minds [91,92].

Second, unlike more traditional forms of political messaging, such as TV and radio advertisements, messages sent to social media users by the platform itself, such as go-vote reminders, are inherently noncompetitive. If these messages were sent in a targeted fashion – say to members of only one political party – the opposing party would have no way to counteract them.

Third, go-vote reminders are ephemeral – impacting people and then disappearing without leaving a paper trail. This makes PME especially dangerous because even if a few people began to suspect that go-vote reminders were being sent in a targeted fashion, both users and authorities would lack the evidence they need to prove that a manipulation has occurred. Without monitoring systems in place, there is no way to tell whether social media platforms are using such content to influence people’s decisions.

We make no specific claims in the present paper about how or whether social media platforms might be using go-vote reminders as a targeted vote-manipulation, but we note that, since 2016, our research group has been developing increasingly larger and more comprehensive systems for preserving online political content being sent to registered US voters on seven online platforms [81]. As of this writing (July 8, 2025), our monitoring system has preserved more than 125 million instances of personalized, ephemeral, online content being sent by Big Tech companies to the computers of more than 16,000 registered voters in all 50 US states. Even if the executives or employees of large social media companies intend to use go-vote reminders only as a public service, the potential that such reminders have to manipulate elections is, in our view, reason enough to study and quantify such a manipulation with some urgency.

## Supporting information

S1 Text Estimating the impact of PME on votes. (DOCX)

S2 Text References for S1 Text. (DOCX)

S1 Fig Bond et al. (2012) go-vote reminders. Reprinted from [7] under a CC BY license, with permission from the corresponding author, James Fowler, original copyright 2012.
(DOCX)

S2 Fig Informed consent form. (DOCX)

S3 Fig Background information on special elections and bond referendums. (DOCX)

S4 Fig Doodlebook instructions. (DOCX)

S5 Fig Screenshot of register-to-vote reminder sent by Facebook in 2024. (DOCX)

S6 Fig Screenshot of go-vote reminder sent by Instagram in 2024. (DOCX)

S7 Fig Post-manipulation debrief. (DOCX)
