# Supplementary material for: The Platform Messaging Effect (PME): A quantification of how go-vote reminders on social media platforms can influence voting intentions
Source: PLoS One. 2026 Mar 2;21(3):e0343692. doi: 10.1371/journal.pone.0343692 (PMC12952607; doi:10.1371/journal.pone.0343692)
Supplement: S1 Text — (DOCX) [file pone.0343692.s001.docx]

**S1 Text. Estimating the impact of PME on votes.**

For estimation purposes, let us first assume that all other sources of influence besides PME – content from television, radio, the internet, newspapers, and magazines, for example – are highly competitive and roughly cancel each other out; they certainly have the potential to do so. Let us further assume that no sources of influence exist to counteract the influence of go-vote reminders on a popular social media platform.

Let us also assume that 6 months before a national election, at least 20% of voters are undecided. Voter surveys conducted before US Presidential elections since the 1940s suggest that the actual percentage probably varies between 30% and 60% [1-3]. In the 2024 Presidential election, 154 million people voted for president [4], which suggests that 6 months before Election Day at least 30 million people (using our conservative 20% estimate) could have been tipped one way or another by an effective, non-competitive source of influence.

Let us further assume that between 60% and 70% of these people sometimes use Facebook (the actual proportion is probably at the high end of this range, if not higher, and that proportion increases from one election to the next worldwide) [5, 6]. Using the more conservative 60% to 70% range, that gives us between 18 and 21 million people to influence with our biased go-vote reminders.

Our study suggests that go-vote reminders in a Facebook feed could increase voting intentions by 14.9%. If, over a 6-month period, we are able to shift 14.9% of our undecided voters, as we did in our experiment, that means that in our election with 154 million voters, we should be able to use go-vote reminders to increase the total number of voters in an election by an additional 2.68 and 3.13 million votes.
