# Supplementary material for: The Platform Messaging Effect (PME): A quantification of how go-vote reminders on social media platforms can influence voting intentions
Source: PLoS One. 2026 Mar 2;21(3):e0343692. doi: 10.1371/journal.pone.0343692 (PMC12952607; doi:10.1371/journal.pone.0343692)
Supplement: S2 Text — (DOCX) [file pone.0343692.s002.docx]

**S2 Text. References for S1 Text.**

1. Annenberg Public Policy Center. 2008 National Annenberg Election Survey Telephone and Online Data Sets. 2010 Dec 8 [Cited 2025 Jul 9]. Available from: <https://www.annenbergpublicpolicycenter.org/2008-naes-telephone-and-online-data-sets/>
2. Liu Y, Ye C, Sun J, Jiang Y, Wang H. Modeling undecided voters to forecast elections: From bandwagon behavior and the spiral of silence perspective. Int J Forecast*.* 2021; 37(2): 461–483. doi: 10.1016/j.ijforecast.2020.06.011.
3. Mayer W. G. The swing voter in American politics. Brookings Institution Press; 2008. pp. 115–119.
4. US Census Bureau. 2024 Presidential election voting and registration tables now available. 2025 Apr 30 [Cited 2025 Jul 9]. Available from: <https://www.census.gov/newsroom/press-releases/2025/2024-presidential-election-voting-registration-tables.html>
5. Schaeffer K. 5 facts about how Americans use Facebook, two decades after its launch. Pew Research Center. 2024 Feb 2 [Cited 2025 Jul 9]. Available from: <https://www.pewresearch.org/short-reads/2024/02/02/5-facts-about-how-americans-use-facebook-two-decades-after-its-launch/>
6. Statista. Number of Facebook users in the United States from 2019 to 2028. 2024 Dec 12 [Cited 2025 Jul 9]. Available from: <https://www.statista.com/statistics/408971/number-of-us-facebook-users/>
