# Supplementary material for: The Platform Messaging Effect (PME): A quantification of how go-vote reminders on social media platforms can influence voting intentions
Source: PLoS One. 2026 Mar 2;21(3):e0343692. doi: 10.1371/journal.pone.0343692 (PMC12952607; doi:10.1371/journal.pone.0343692)
Supplement: S1 Fig — Reprinted from [7] under a CC BY license, with permission from the corresponding author, James Fowler, original copyright 2012. (DOCX) [file pone.0343692.s003.docx]

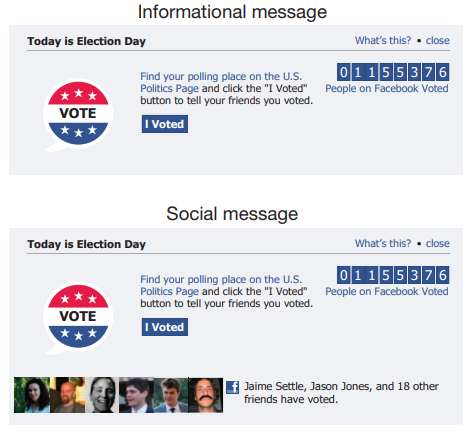


**S1 Fig. Bond et al. (2012) go-vote reminders.** Reprinted from [7] under a CC BY license, with permission from the corresponding author, James Fowler, original copyright 2012.
