# Supplementary figures and images for: The Platform Messaging Effect (PME): A quantification of how go-vote reminders on social media platforms can influence voting intentions
Source: PLoS One. 2026 Mar 2;21(3):e0343692. doi: 10.1371/journal.pone.0343692 (PMC12952607; doi:10.1371/journal.pone.0343692)

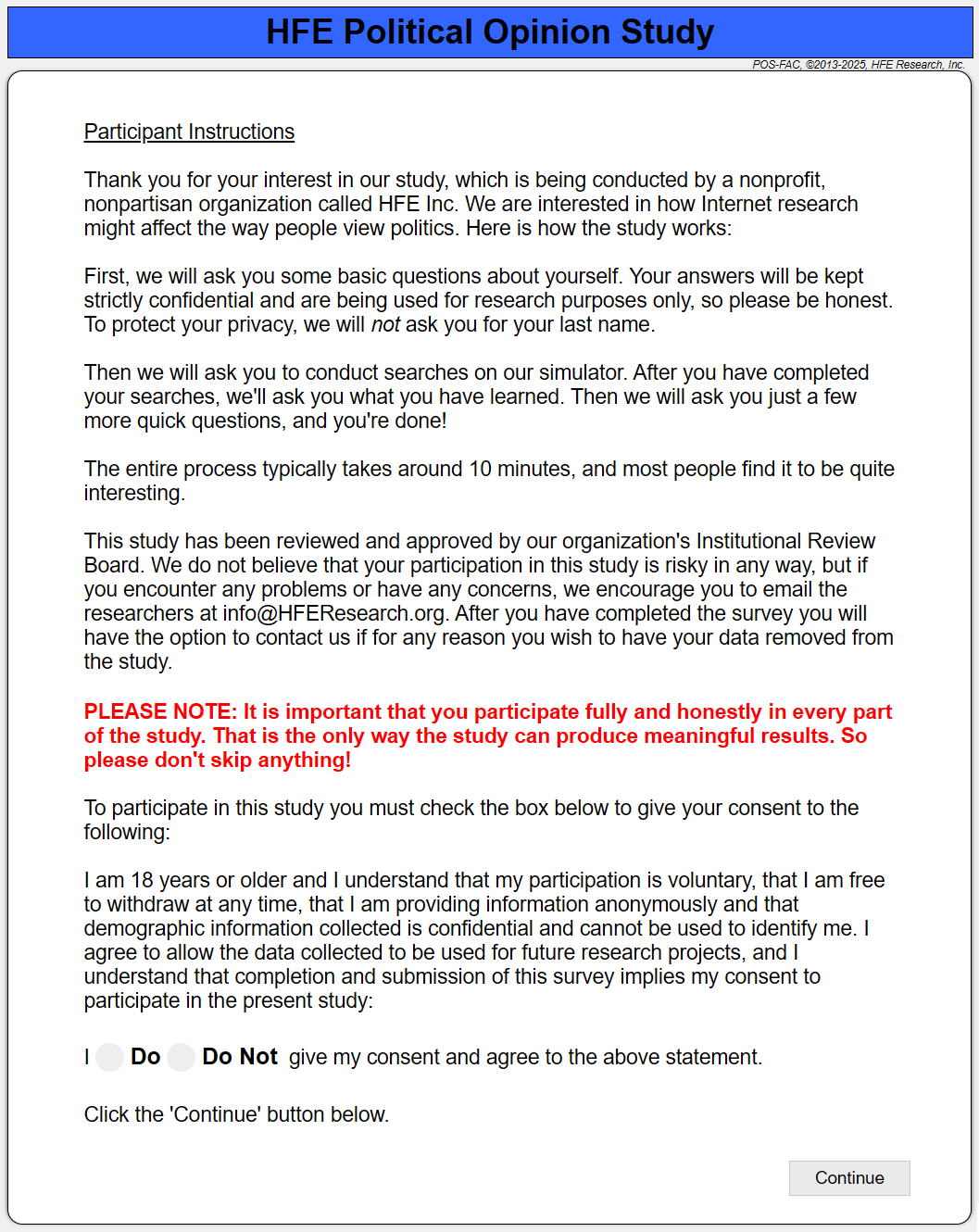


**S2 Fig. Informed consent form.**

Supplement: S2 Fig — (DOCX) [file pone.0343692.s004.docx]

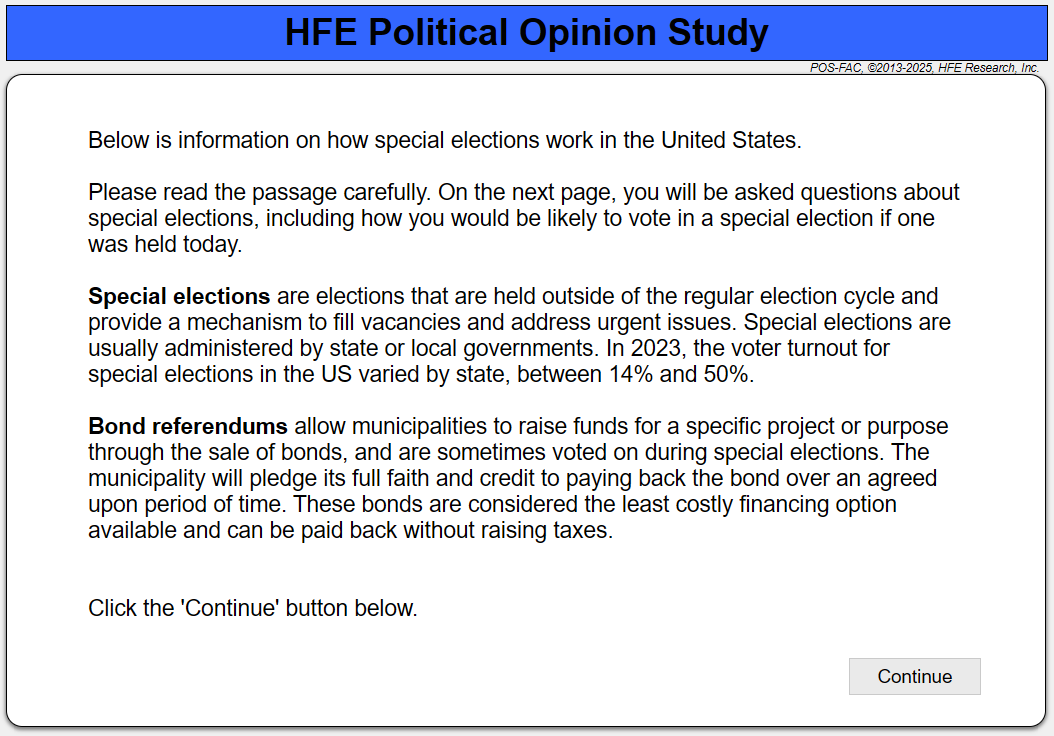


**S3 Fig. Background information on special elections and bond referendums.**

Supplement: S3 Fig — (DOCX) [file pone.0343692.s005.docx]

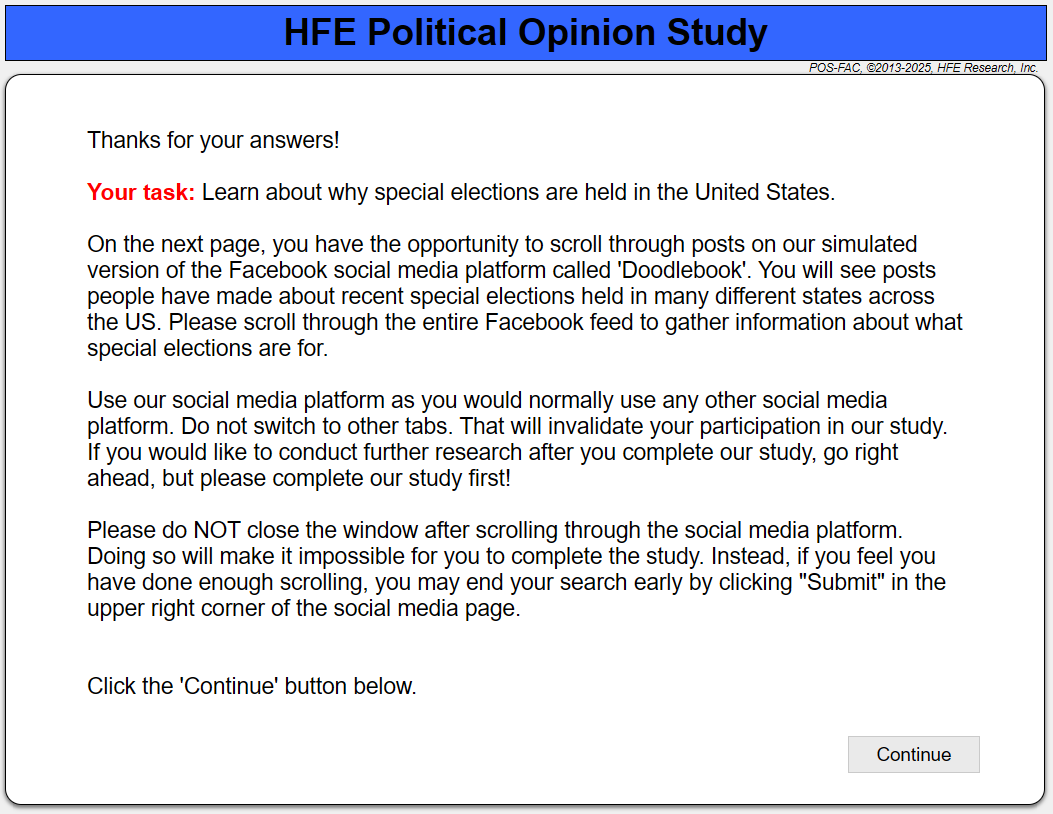


**S4 Fig. Doodlebook instructions.**

Supplement: S4 Fig — (DOCX) [file pone.0343692.s006.docx]

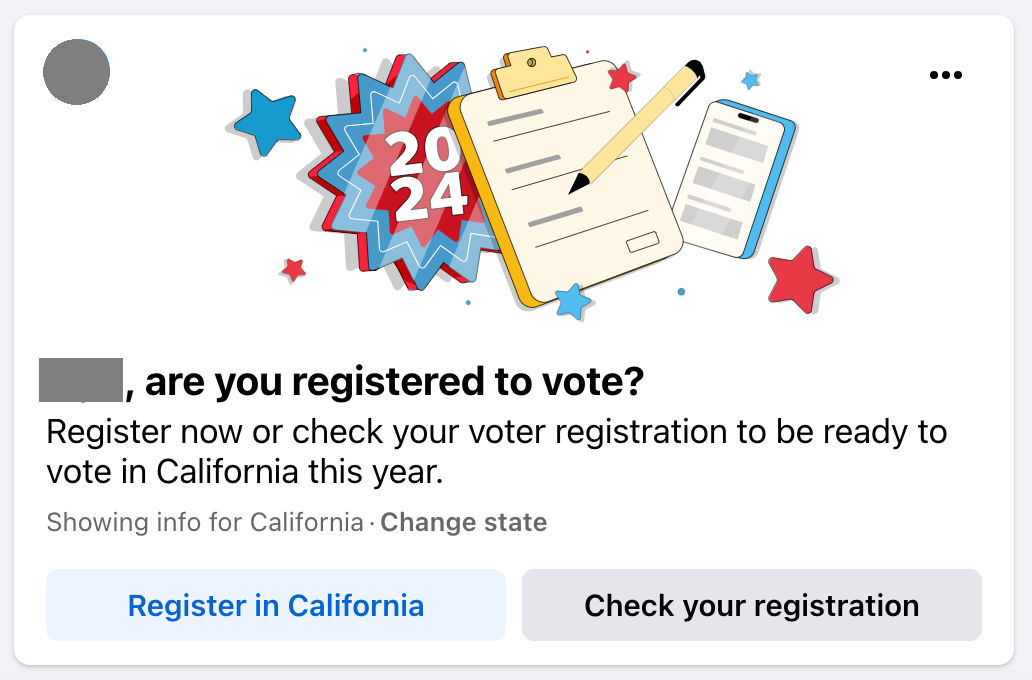


**S5 Fig. Screenshot of register-to-vote reminder sent by Facebook in 2024.**

Supplement: S5 Fig — (DOCX) [file pone.0343692.s007.docx]

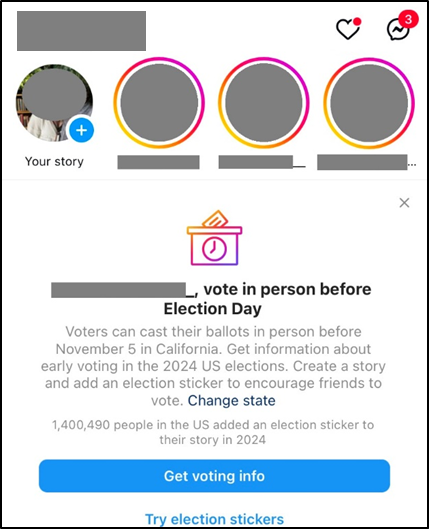


**S6 Fig. Screenshot of go-vote reminder sent by Instagram in 2024.**

Supplement: S6 Fig — (DOCX) [file pone.0343692.s008.docx]

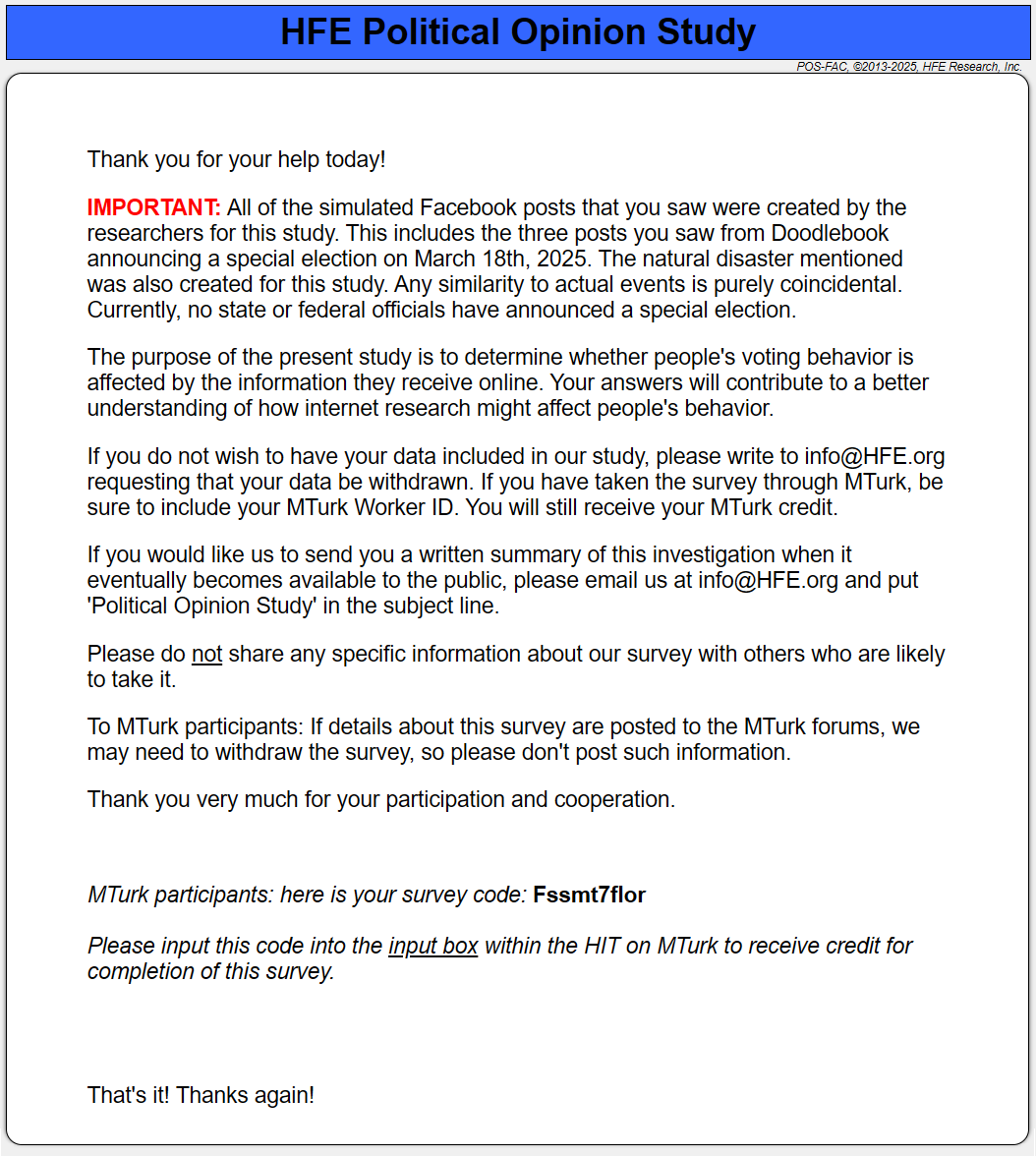


**S7 Fig. Post-manipulation debrief.**

Supplement: S7 Fig — (DOCX) [file pone.0343692.s009.docx]
